# Predictions of Cu, Zn, and Cd Concentrations in Soil Using Portable X-Ray Fluorescence Measurements

**DOI:** 10.3390/s20020474

**Published:** 2020-01-14

**Authors:** Karl Adler, Kristin Piikki, Mats Söderström, Jan Eriksson, Omran Alshihabi

**Affiliations:** Department of Soil and Environment, Swedish University of Agricultural Sciences, SE-75007 Uppsala/SE-53223 Skara, Sweden; kristin.piikki@slu.se (K.P.); mats.soderstrom@slu.se (M.S.); jan.o.eriksson@slu.se (J.E.); omran.alshihabi@slu.se (O.A.)

**Keywords:** PXRF, soil, copper, zinc, cadmium, machine learning, precision agriculture

## Abstract

Portable X-ray fluorescence (PXRF) measurements on 1520 soil samples were used to create national prediction models for copper (Cu), zinc (Zn), and cadmium (Cd) concentrations in agricultural soil. The models were validated at both national and farm scales. Multiple linear regression (MLR), random forest (RF), and multivariate adaptive regression spline (MARS) models were created and compared. National scale cross-validation of the models gave the following R^2^ values for predictions of Cu (R^2^ = 0.63), Zn (R^2^ = 0.92), and Cd (R^2^ = 0.70) concentrations. Independent validation at the farm scale revealed that Zn predictions were relatively successful regardless of the model used (R^2^ > 0.90), showing that a simple MLR model can be sufficient for certain predictions. However, predictions at the farm scale revealed that the non-linear models, especially MARS, were more accurate than MLR for Cu (R^2^ = 0.94) and Cd (R^2^ = 0.80). These results show that multivariate modelling can compensate for some of the shortcomings of the PXRF device (e.g., high limits of detection for certain elements and some elements not being directly measurable), making PXRF sensors capable of predicting elemental concentrations in soil at comparable levels of accuracy to conventional laboratory analyses.

## 1. Introduction

Mapping concentrations of micronutrients or toxic elements in agricultural soil is important but is not commonly done. This kind of information could be useful in precision agriculture, where the goal is optimal management in space and time [1]. For instance, zinc (Zn) and copper (Cu) are important elements in crop production due to their roles in photosynthesis, respiration, and other plant functions [2,3]. However, excessively high concentrations can be toxic for crops (e.g., an excessive concentration of Cu can lead to malformation of root systems) [3]. Hence, there is a need to detect both low and high concentrations. Cadmium (Cd) is also toxic to consumers of crop products above certain threshold concentrations [2]. Thus, it can be useful to map Zn, Cu, and Cd at the field scale in order to rectify deficiencies and toxicities, and to safeguard crop quality and food safety. At present, there are no public field-scale maps of these elements in Sweden.

In Sweden, deficiency of Cu in crops is known to occur in sandy and organic soils [4], whereas availability of Zn is regarded as less of a problem. However, Zn deficiency in agricultural soil is a common problem in many other parts of the world [5]. Very high concentrations of Cd are typically related to the soil’s parent material, which can vary substantially within an agricultural field [6]. In Sweden, a soil is deemed to be at risk of Cu deficiency at concentrations below 6–8 mg kg^−1^ [7]. There are no regulations governing Cd concentration in agricultural soil, but there are national laws that prohibit application of sewage sludge when soil concentrations are above the stated limits for Cu (40 mg kg^−1^), Zn (100–150 mg kg^−1^), and Cd (0.4 mg kg^−1^) [7,8].

To derive accurate maps of elemental concentrations in soil, many soil samples need to be analyzed. The conventional method involves element extraction with acids followed by analysis using the inductively coupled plasma (ICP) technique [9,10]. However, wet chemistry laboratory analyses can be expensive, time-consuming, and destructive to the sample [9,10]. The portable X-ray fluorescence (PXRF) technology is becoming an interesting option as it is a cheap, fast, and non-destructive method for analyzing element concentrations in soil samples [11]. This makes it very suitable for tasks where high sampling density is needed (e.g., mapping and geostatistics) [12]. The method works by exciting atoms with an energy source from the PXRF device, often an X-ray tube [13]. The atoms then emit X-ray fluorescence at specific wavelengths depending on the element in question, which is then measured by a sensor in the PXRF device [13]. The method can be accurate when combined with a simple preparation of the soil sample, and can provide high-quality data comparable to those obtained with conventional methods for quantification of certain elements in soil samples [14]. The PXRF technology is recognized as an official method for analyzing trace elements in soil by the United States Environmental Protection Agency (U.S. EPA) [15].

The aims of the present study were to:

- Use PXRF measurements to create national models for prediction of soil Cu, Zn, and Cd concentrations in agricultural soils;
- Validate these models at the national scale using cross-validation, and at the farm scale using an independent dataset;
- Compare the performance of three model types: multiple linear regression (MLR), multivariate adaptive regression splines (MARS), and random forest regression (RF);
- Test whether the best model for Cu can accurately predict whether a sample has concentrations above or below recommended levels;
- Test whether the best model for each element can accurately predict whether a soil sample has Cu, Zn, and Cd concentrations above or below the permissible level for sewage sludge application to agricultural soil.

## 2. Materials and Methods

### 2.1. Soil Sampling

**Figure 1:**
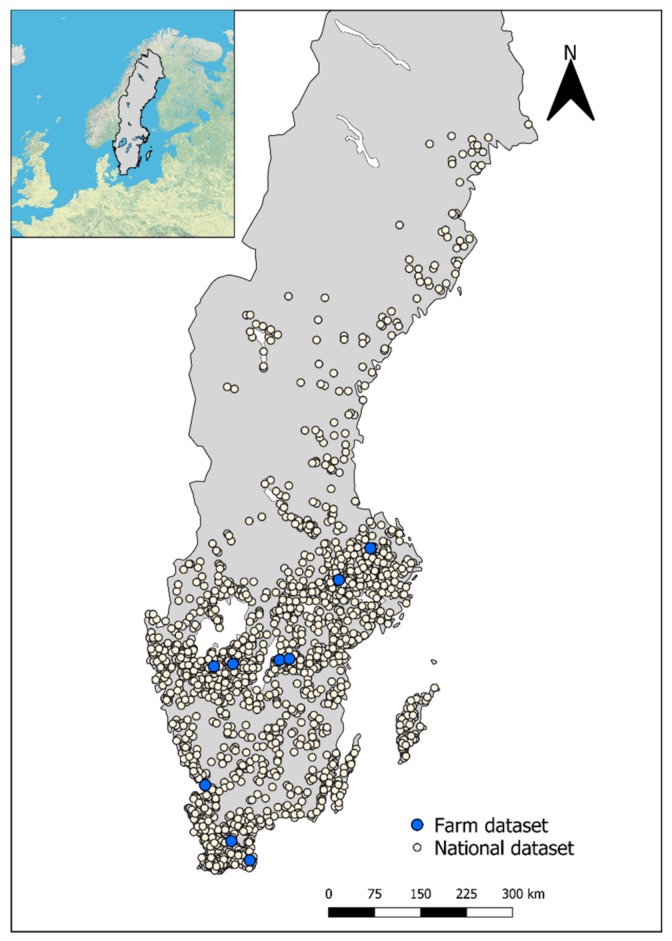
Map of Sweden showing soil sampling locations used in the present study. Farm dataset refers to the nine farms that were used for independent validation of the models. National dataset refers to the calibration samples. Base map courtesy of Environmental Systems Research Institute (ESRI) (Redlands, CA, USA).

**Figure 2:**
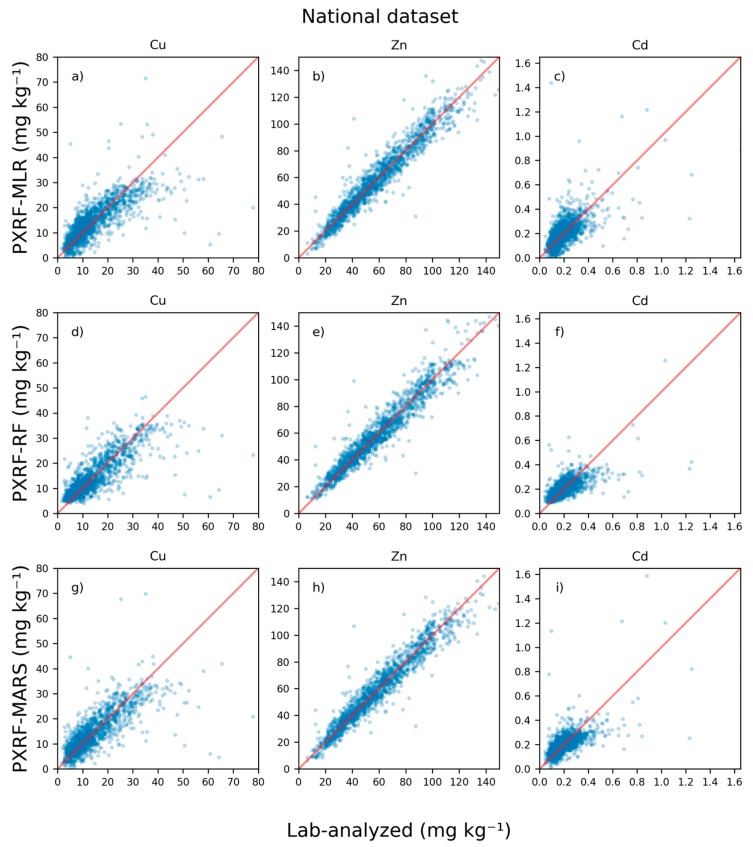
Concentrations of copper (Cu), zinc (Zn), and cadmium (Cd) predicted from portable X-ray fluorescence (PXRF) measurements using multiple linear regression (MLR), random forest regression (RF), and multivariate adaptive regression splines (MARS) for national-scale data using leave-one-out cross-validation compared with 7M HNO_3_ extraction and inductively coupled (ICP) analysis. The symbols are semi-transparent to show point density.

The study area included all agricultural land in Sweden. Swedish crop production (mostly small-grain crops, oilseeds, pastures, and meadows covering about 2.5 Mha) is mainly concentrated in young, marine, and lacustrine post-glacial sediments from the time after the Weichselian glaciation [16]. More than 90% of the agricultural area is located in the southern area of the country (the sample distribution in Figure 1 accurately depicts the occurrence of arable land). Eutric and dystric cambisols are the dominant cropland soil types [16]. Cropland soil texture ranges from heavy clays in the eastern parts, to loam and sandy loam generally dominating in the south and southwestern agricultural areas [16,17,18]. For a general soil and texture map of Sweden, see Figures 2 and 4 by Eriksson et al. [19]. For an overview of topsoil properties of arable land in Sweden, see maps by Eriksson et al. (pp. 75–90) [7]. Descriptive statistics of the soil properties in the calibration samples are presented in Table 1.

The total number of topsoil samples available from the national monitoring program for arable soils in Sweden was 1833 [7]. Sampling locations in the monitoring program were selected using a random stratified sampling design covering all arable land [7]. Soil samples from nine farms (n = 179, ~ 20 from several fields per farm) were used for validation at the farm scale (Figure 1). The nine farms were originally selected for a previous study in order to represent a wide range of Cd concentrations and different geologies, based on maps presented in Eriksson et al. [7]. Each soil sample consisted of nine subsamples collected with an auger at a depth of 0–20 cm within a 3–5 m radius of the sample coordinates. The soil samples were air-dried, homogenized, and sieved (< 2 mm) prior to analysis.

### 2.2. PXRF Measurements

The soil samples were analyzed ex situ using a Niton XL3t GOLDD+PXRF device with a geometrically optimized large area drift detector and an Ag anode that operates at 50 kV and 200 µA (Thermo Scientific, Billerica, MA, USA), which were connected to a computer and mounted on a static frame specially designed for the PXRF device (Thermo Scientific, Billerica, MA, USA). The PXRF device was set in “soil mode”, an instrument-specific measurement configuration optimized for soil materials, and measurement time was set to 180 s per sample. Each soil sample was dried, homogenized, and sieved (< 2 mm) according to recommendations for ex situ PXRF analysis [13,15]. Each soil sample was placed in a 32-mm double-ended XRF sample cup (filled to three-quarters volume) with a 4-µm thick transparent polypropylene XRF film in line with U.S. EPA standards [15] and placed on the PXRF aperture. The reference standard 2709a from the National Institute of Standards and Technology (NIST) was measured four times during the project to check the measurement stability of the PXRF device (see Supplementary Materials). Measurements were found to be stable over the course of the project.

The limit of detection (LOD) was set at three times the standard deviation of the measurement. The PXRF device measured each second for the duration of measurement (180 s). Hence, a final concentration and standard deviation were provided for the element in question when the measurement was completed. As each measurement has its own individual standard deviation for an element, there is no common LOD for an element. Measured values below this limit were denoted “not a number” (NaN). Only elements with < 10% NaN values in the national dataset were included in the modelling to ensure that the measured concentrations of elements used as explanatory variables were generally above the LOD of the PXRF device. Hence, future measurements with a similar PXRF device can be used with high probability. All samples that exhibited NaN values for any of the included elements were excluded from the modelling. The total number of samples used for calibration was 1520 (Soil properties of these samples can be seen in Table 1).

### 2.3. Laboratory Analyses

Pseudototal concentrations of Cu, Zn, and Cd in the soil samples were determined by extraction with 7M HNO_3_ in an autoclave at 120 °C for 30 min, as stated by Swedish standard SS 28 31 11 [20]. Measurement was performed using inductively coupled plasma atomic emission spectroscopy (ICP-AES) for Zn and Cu, and inductively coupled plasma mass spectrometry (ICP-MS) for Cd. Hereafter, “lab-analyzed” refers to results obtained with this extraction and analysis method.

### 2.4. Modelling

#### 2.4.1. Model Selection

Three different machine learning algorithms were chosen for modelling Zn, Cu, and Cd concentrations, namely MLR, RF, and MARS. The intention was to have a simple linear model (MLR) and two distinct non-linear models (RF and MARS). The RF and MARS algorithms produce non-linear models with discrete and continuous predictions respectively. RF consists of an ensemble of decision trees with bagging, where each decision tree is made from a partitioning algorithm based on conditional statements. The term bagging means creating several decision trees from different subsets of the data, making the final predicted value the mean value of several tree models [21]. MARS is based on building several piecewise linear regression models (basis functions) that are valid within certain intervals of the explanatory variables and defined by hinge functions [21]. The MARS algorithm first creates basis functions in a forward pass, later to be pruned in a backwards pass to reduce model complexity and risk of overfitting [21]. For a more detailed description of MLR, RF, and MARS, see Hastie et al. [21].

#### 2.4.2. Model Implementation

The MLR and RF algorithms were implemented using the Scikit-learn machine learning package (version 0.19.1) for Python [22]. MARS was implemented using the Py-earth package (version 0.1.0) for Python, originally made for the R programming language [23]. Both RF and MARS were used in their default setting. For example, MARS was set as default to be additive. This was done to reduce overfitting and make the models more robust. The only hyperparameter set was with the RF models, as the number of bagged trees needed to be specified (number of trees was set to 100). For a complete description of the default settings, see the respective model descriptions in the Py-earth and Scikit-learn packages. Predictions of negative concentrations were set to 0 mg kg^−1^.

#### 2.4.3. Validation

The performance metrics used were the mean absolute error (MAE) and the coefficient of determination (R^2^), often named the Nash–Sutcliffe model efficiency coefficient [24], which is defined as in Equation (1):

(1) $$R^{2}=1-\frac{\sum {\left(y_{i}-{\hat{y}}_{i}\right)}^{2}}{\sum {\left(y_{i}-\bar{y}\right)}^{2}}$$

where *y_i_* is the actual value, *ŷ_i_* is the predicted value, and $\bar{y}$ is the mean of the actual values of the response variable. 

Cross-validation was performed on the national dataset using the leave-one-out method for each MLR, RF, and MARS model. Validation metrics are also presented based on how well the Cu and Cd models performed at lower concentrations (arbitrarily chosen range of interest (ROI)) of 0–20 mg kg^−1^ and 0–0.5 mg kg^−1^, respectively. This was done to generate validation statistics that give a better understanding of how well the predictions perform around concentrations of practical interest. 

In addition, confusion matrices were created to assess whether the models could be used to determine if a soil element concentration is above or below a given threshold in the cross-validation for Cu deficiency and sewage sludge application with regard to Cu, Zn, and Cd concentrations. The upper boundary of the Cu deficiency threshold was used (8 mg kg^−1^). The models chosen for this task were those that performed best in terms of R^2^ in the cross-validation for each element. Agreement of the predictions was calculated according to Equation (2):

(2) $$Agreement=\frac{\left(Tp+Tn\right)}{\left(Tp+Tn+Fp+Fn\right)}$$

where *Tp* is the total number of positive predictions, *Tn* is the total number of negative predictions, *Fp* is the number of false positive predictions, and *Fn* is the number of false negative predictions. Positive predictions refer to values below the threshold and negative predictions refer to values above the threshold.

## 3. Results

### 3.1. Descriptive Statistics of PXRF Measurements of the National Set of Soil Samples

Thirteen elements proved to be useful as explanatory variables of element concentrations in the 1833 samples (Table 2). The element closest to the threshold of < 10% NaN readings was Cs (9.8% NaN), followed by barium (Ba) (3.9%), lead (Pb) (2.2%), vanadium (V) (1.4%), manganese (Mn) (0.4%), and Zn (0.2%). The remaining elements shown in Table 2 had no NaN readings. Descriptive statistics of the elements used to calibrate the MLR, RF, and MARS models are also presented in Table 2. The descriptive statistics minimum, maximum, mean, median, and standard deviation (SD) were calculated after removal of samples with NaN values in any of the included variables, which resulted in exclusion of 313 samples out of the original 1833 samples (i.e., 1520 samples were used for modelling). The majority of the samples excluded had readings below the LOD for Cs.

A total of 99% of Cd measurements and 55% of Cu measurements were NaN, indicating that this PXRF device cannot be used for direct measurement of Cd and Cu at the concentration range found in Swedish agricultural soil. The lowest concentration of Cu measured was approximately 20 mg kg^−1^, indicating that this is perhaps the lowest possible Cu concentration that can be measured with this PXRF device.

The PXRF device measured values similar to known concentrations of the included elements in NIST 2709a (Table 2). Concentrations of some elements, such as Cs and Pb, were overestimated and underestimated, respectively. However, the stability of the measurements, as shown by the standard deviation of the recovery rates, shows that the PXRF measurements can be used for modelling, as the coefficients in the calibrated models will be valid over time. Measurements of Cs had the least stability according to the standard deviation of the recovery rates, but still only fluctuated by 1–3 mg kg^−1^ (see Supplementary Materials).

### 3.2. Descriptive Statistics of the National and Farm Datasets

Descriptive statistics of lab-analyzed Cu, Zn, and Cd concentrations for the national dataset (calibration and cross-validation data) and the farm dataset (validation data) are shown in Table 3.

The national and farm datasets differed in their frequency distributions of concentrations of Cu, Zn, and Cd. The mean and median showed that the farm dataset generally had higher concentrations of Cu, Zn, and Cd than the national dataset. For example, the national dataset contained five samples with Cu concentrations above 60 mg kg^−1^, while the farm dataset contained 19. Similarly, eight samples in the national dataset had Cd concentrations above 1 mg kg^−1^, while there were 25 such samples in the farm dataset. There were, therefore, more samples with higher concentrations of Cu and Cd in the farm dataset than in the national dataset, even though the farm dataset was much smaller. In the national dataset, high concentrations were, therefore, less common in the case of Cu and Cd. For Zn there were 139 samples with concentrations above 100 mg kg^−1^ in the national dataset, while there were 41 in the farm dataset. This implies that the Zn concentrations measured on the selected farms were more similar to those in the national dataset than the measured concentrations of Cu and Cd

### 3.3. Cross-Validation

In Figure 2, cross-validated leave-one-out predictions of concentrations from the MLR, RF, and MARS models for each element are plotted against lab-analyzed concentrations for the national dataset. The cross-validation results showed that it was possible to predict concentrations beneath the LOD for Cu, which was approximately 20 mg kg^−1^. However, the RF models could not predict concentrations as low as those predicted by the continuous MLR and MARS models, as is apparent for Cu and Cd predictions with the RF models (Figure 2). For instance, the RF model for Cu could only predict concentrations down to approximately 5 mg kg^−1^, while the MLR and MARS models could predict lower concentrations. There was no major visual difference between the performance of the MLR and MARS models for Cu (Figure 2). All three models for Zn imposed a fit close to the 1:1 line. The MLR model for Cd produced errors in the higher range of concentrations, while the RF and MARS models predictions at higher concentrations exhibited negative bias. However, the MARS model for Cd gave smaller errors at lower concentrations than the MLR model for Cd (Table 4).

**Figure 3:**
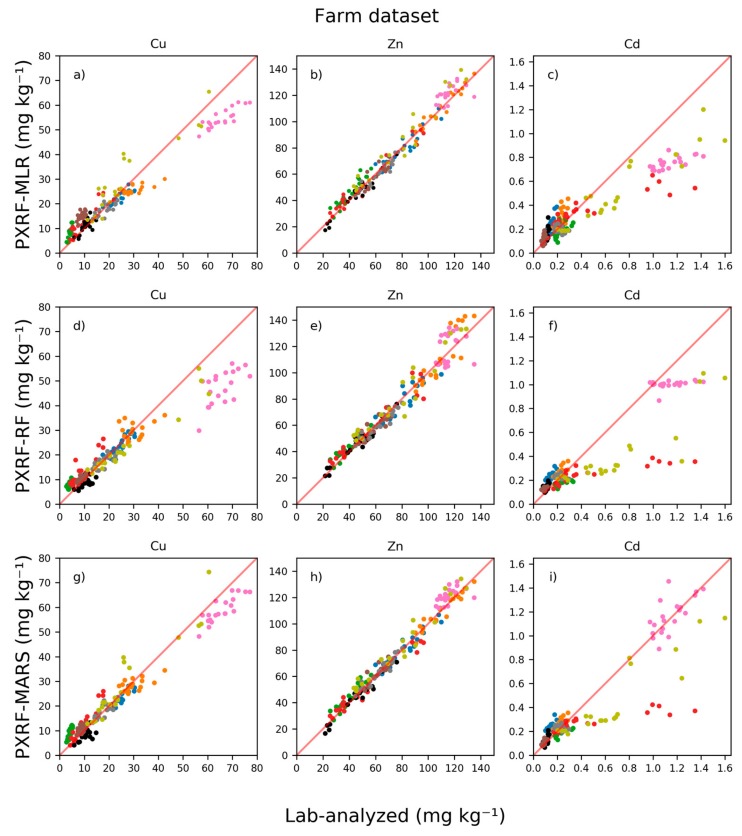
Concentrations of copper (Cu), zinc (Zn), and cadmium (Cd) predicted from portable X-ray fluorescence (PXRF) measurements using multiple linear regression (MLR), random forest regression (RF), and multivariate adaptive regression splines (MARS) on the farm dataset compared with 7M HNO_3_ extraction and inductively coupled (ICP) analysis. The models were calibrated at the national scale and applied on the farm dataset. Each color represents a specific farm.

For ease of comparison, the same range of values is shown in the farm-scale validation (Figure 3) and in the national-scale validation (Figure 2). This means that some values outside the range are not shown in Figure 2 (1, 20, and 5 values for Cu, Zn, and Cd, respectively).

Validation statistics revealed that the MARS models generally performed best except for with Cu, for which the RF model performed best, based on R^2^ and MAE (Table 4). Cross-validation revealed two problems with the continuous models MLR and MARS, especially in the ROI for Cu and Cd. The first was the impact on strange predictions of concentrations for certain samples (e.g., in terms of R^2^, the MLR model for Cu exhibited little accuracy). However, removal of a single poorly predicted sample resulted in an increase in R^2^ from 0.06 to 0.13. For the MLR model for Cd, R^2^ increased from −0.17 to 0.01 with removal of the same sample. The second problem involved predictions below 0 mg kg^−1^. The numbers of samples with predicted concentrations below 0 mg kg^−1^ were 10 and 8 for the MLR and MARS models for Cu, respectively. The numbers of samples with Cd predictions below 0 mg kg^−1^ were 12 and 1, respectively, for the MLR and MARS models. Hence, predictions below 0 mg kg^−1^ were uncommon. In the ROI, there were 1196 and 1487 samples for Cu and Cd, respectively.

These problems of predictions of outlier samples and predicted negative concentrations were not observed with the discrete predictions of the RF model, as it cannot extrapolate beyond the calibration data.

### 3.4. Validation at the Farm Scale

The MLR, RF, and MARS models of concentrations for each element in the farm-scale validation are compared with the lab-analyzed concentrations in Figure 3, where each farm is represented by a specific color (see full equations for the MLR models in the Supplementary Materials). Validation statistics are shown in Table 5. All models were able to predict below the LOD of the PXRF device for Cu, as also seen in the cross-validation. At lower concentrations, the MLR model for Cu had a general positive bias, while at higher concentrations it had a general negative bias. The RF and MARS models for Cu also exhibited negative bias for predictions at higher concentrations, with MARS having the least negative bias. However, at lower concentrations the RF and MARS models exhibited less positive bias in predictions than the MLR model. Farms with lower concentrations showed a smaller spread in predicted values with the RF model for Cu (i.e., the farms represented by green symbols) compared with the MLR and MARS models (Figure 3). However, as in the cross-validation, the RF model could not predict concentrations as low as those predicted by the MLR and MARS models for Cu, which resulted in a positive bias in predictions for farms with low concentrations of Cu. All models, though especially the MARS model, were able to predict variations in Cu concentrations on certain farms with ranges of Cu concentrations.

The MLR model for Zn was able to predict throughout the range with relatively high accuracy and perhaps a slight positive model bias. The MLR model for Cd showed similar problems to the MLR model for Cu, as high concentrations could not be predicted and there were errors in prediction at lower concentrations. In general, all models showed equally good performance, with more or less bias in the predictions in some cases.

The MLR and RF models for Cd exhibited positive bias in the predictions at lower concentrations, while the MARS model exhibited the least positive bias. However, as can be seen from Figure 3, three specific farms were difficult to predict. One farm, colored red, had Cd concentrations ranging from about 0.5 to 1.4 mg kg^−1^. The Cd concentrations on this farm could not be accurately predicted by any model tested in this study. However, sites on the farm exhibiting the highest concentrations of soil Cd, colored pink, were those most accurately predicted by the MARS model.

Validation metrics of the prediction at the farm scale are presented in Table 5. These include validation metrics on how well the Cu and Cd models performed in the ROI, for which there were 102 and 140 samples for Cu and Cd, respectively.

Based on the metrics, the nationally calibrated MARS models performed best of the models tested in the farm-scale validation for Cu, Zn, and Cd (Table 5). However, within the ROI the RF model for Cu performed better than the MARS model for Cu. This can also be inferred from Figure 3, where predictions for each farm with lower concentrations showed less spread. The MARS model performed better for the whole range than the RF model for Cu. All the models for Zn at the farm scale performed very similarly, but with the MARS model the performance was the best, as also observed in the cross-validation. Using the Zn values measured on the farms with the PXRF device compared with lab-analyzed values resulted in R^2^ = 0.81, which was lower than that of the MLR, RF, and MARS models for Zn (Table 5). The best model for predicting Cd, for the whole range and within the ROI, was the MARS model, as also found in the cross-validation.

### 3.5. Testing Performance for Fertilization and Sewage Sludge Fertilization

Confusion matrices of predictions in relation to actual concentrations above or below threshold concentrations for Cu, Zn, and Cd in the cross-validation are presented in Table 6. The thresholds are based on the recommendations for Cu fertilization and permissible levels of soil Cu, Zn, and Cd concentrations, above which sewage sludge application is prohibited [4,7]. The models used were those identified as the best based on the coefficient of determination, presented in Table 4. Thus, the RF model was used for Cu, MLR was used for Zn, and MARS was used for Cd.

The level of agreement between predicted and lab-analyzed values was 82% when predicting whether a soil was Cu-deficient or not (Table 6). However, there was higher accuracy in predicting soils that were not Cu-deficient in the national dataset (94% correctly classified) compared with those that were Cu deficient (53% correctly classified) (see Figure 2).

Assessment of samples regarding suitability for sewage sludge application revealed high agreement between predicted and lab-analyzed values for Cu, Zn, and Cd (98%, 95%, and 95%, respectively). This was especially true for predictions below the respective threshold. Most of the samples had concentrations below the permissible level for sewage sludge application (shown in Figure 2).

## 4. Discussion

The results in this study demonstrated that an approach based on PXRF measurements coupled with machine learning algorithms is capable of predicting concentrations of Cu, Zn, and Cd in non-organic (SOM < 20%; Table 1) Swedish agricultural soils that can be used for risk assessments. An interesting finding was that concentrations of elements that are difficult or impossible to measure directly with the PXRF device, such as Cu and Cd, can be indirectly predicted with predictor elements present in measurable concentrations in Swedish agricultural soil (shown in Table 2). For example, it was found that MLR modelling of Zn was better than only using direct measurements of Zn made with the PXRF device. However, the relatively accurate results obtained with the MLR model for Zn were attributable to some degree to PXRF-measured Zn being included as an explanatory variable. Cd concentrations were most difficult to predict accurately, as was evident for certain farms in the farm-scale validation, for which medium and high concentrations could not be predicted without substantial errors. Hence, predictions of lower concentrations can be deemed more accurate.

The method presented for creating predictive models from PXRF measurements is a valid option (especially for Cu and Zn) when a dense sampling scheme is needed to create high-resolution maps of Cu, Zn, and Cd showing within-field variation. This can be a powerful tool in precision agriculture and for regional or national soil monitoring and mapping projects.

### 4.1. Cu Deficiency

There are certain ranges of Cu concentrations that are especially interesting for Swedish agriculture. According to Swedish recommendations [4], a soil is deemed to be at risk of Cu deficiency when the concentration is below 6–8 mg kg^−1^. Indications of soil Cu status could be obtained using the MARS and RF models for Cu, where the predictions could be used to assess whether a soil is at risk of being Cu-deficient or not, considering the high model agreement and MAE in the ROI (Table 5 and Table 6). Using PXRF, Hu et al. [14] obtained accurate measurements of Cu comparable to those in laboratory analysis (R^2^ = 0.67). In this study, we achieved substantially higher R^2^ relative to laboratory analysis when predicting Cu (up to R^2^ = 0.94). Hence, using PXRF measurements for prediction appears promising. However, the MAE in the ROI was 2–3 mg kg^−1^ depending on the model used, so predictions around the threshold of 6–8 mg kg^−1^ should be viewed with caution and complementary conventional laboratory analysis should be conducted. For example, it should be noted that the results presented in Table 6 are binary, while the input data for this classification were not. This means that if a sample is predicted to have a concentration of 8.1 mg kg^−1^ (i.e., slightly above the threshold), the prediction will be classified as incorrect. For example, if a predicted sample was deemed to be correctly predicted up to 9 mg kg^−1^, the number of correctly predicted samples increased from 224 to 294 with the RF model for Cu.

### 4.2. Sewage Sludge Application

The prediction models could be used to determine whether sewage sludge may be applied in an agricultural field. For example, the best model for predicting soil Cd concentrations had an MAE of 0.04 mg kg^−1^ in the ROI in the farm-scale validation with the MARS model, which makes it possible to determine whether an agricultural soil is at risk of excessive Cd concentrations. The results showed that Zn predictions were of high accuracy and good model agreement (Table 6). López-Núnez et al. [25] showed a similar high accuracy of predicted Zn in organic amendments with a linear model. Hence, predictions of Zn concentrations with PXRF appear highly suitable. This study showed that the MLR model is sufficiently accurate to predict whether sewage sludge application is permissible in relation to soil Zn concentrations. A similar level of agreement was found in the Cu and Cd predictions. However, most samples in the national dataset had Cu, Zn, and Cd concentrations below the threshold where sewage sludge application is legal. Hence, concentrations above the legal limits can be deemed as outliers in the distribution and the predictions at these concentrations should be viewed with caution. Similar to the results in Table 6 mentioned earlier, there might be a need to perform a conventional laboratory analysis when predicted concentrations are close to the thresholds for sewage sludge application.

### 4.3. Data, Model Selection, PXRF Methodology, and Variable Selection

The MAE in the national cross-validation was generally lower than in the farm-scale validation and the descriptive statistics showed that the farm dataset was somewhat unrepresentative of the national dataset. Thus, the farm-scale dataset can be regarded as rather difficult to predict accurately, since the farms included are quite unique in terms of their high Cd and Cu values (see Table 3). Hence, as shown by the results, predictions of mid- and high-range concentrations of Cd should be viewed with caution. The results indicated that other predictors from other sensors may be needed when there is little variation in concentrations measured by PXRF. However, some farms with varying concentrations of Cu and Cd were predicted with accuracy, which indicates that the farm dataset is difficult to validate against in some cases.

The results showed that when using the models presented, some caution is needed. For instance, the RF models cannot predict concentrations as low as those predicted by the continuous MLR and MARS models. However, in rare instances MARS and MLR can predict non-sensical concentrations. The RF algorithm benefits greatly from a uniform distribution of concentrations in the calibration dataset in order to create classes throughout the range. In the present study, an insufficient number of classes was constructed by the RF model in the higher ranges for Cd. This implies that the accuracy of the RF model could be improved with more samples, so that more variations in soil Cd could be accounted for. Overall, the continuous models tested in this study appear more interesting as they allow more extrapolation in predictions.

A simple linear model such as MLR can be very effective, as seen with the predictions of Zn, and in some instances Cu. Non-linear models such as RF and especially MARS can be better overall options, as there is lower associated error in the predictions. Hence, depending on the range of concentrations to be predicted, either RF or MARS might be more or less suitable. For example, the RF model for Cu performed the best out of the Cu models at lower concentrations, but was unable to makes predictions as accurately as the MARS model at higher concentrations.

It should be noted that the PXRF measurements and the models were made on processed samples. This means that these models might not be appropriate when using PXRF in the field due to the sensitivities of the method to soil matrix factors, such as moisture and particle size distribution [26]. The parameterized models are, thus, calibrated for a specific soil matrix type, which in the present case was dry, homogeneous, and fine-grained soil. The PXRF device was used as a small, nimble laboratory device that is easy to use and provides ample amounts of data in terms of measured elements, in a shorter time, and at a lower cost than conventional laboratory analysis, even when used in an ex situ setting [14,27]. Based on the results in this study, the method will be tested in the future on a larger dataset of soil samples to create maps of the modelled elements. Hence, this study provides excellent groundwork for a future where these models are the foundation in mapping of soil Cu, Zn, and Cd concentrations in Sweden.

In the present study, no feature selection of predictor elements was performed. This was because with national models, the relationship between predictor elements and the target element in question can vary in space. For instance, when the present analysis was performed with half the dataset, particular relationships between elements were more prevalent, while these relationships were not present when the whole dataset was used. However, if regional models are to be created in future studies, feature selection might be necessary, as certain relationships depend on the soil type and underlying geology.

## 5. Conclusions

- Predictive models using PXRF measurements were created and found to be applicable at farm and national scales;
- The models were able to predict concentrations of Cu, Zn, and Cd in non-organic Swedish agricultural soils at both national and farm levels, but with varying amounts of error;
- Non-linear models proved most suitable for predicting concentrations of Cu and Cd, while the linear model for Zn yielded predictions with the same level of accuracy as the non-linear models;
- The accuracy of predictions means that the models created can be used to assess the risk of Cu deficiency. However, complementary laboratory analysis is advisable if predicted concentrations are close to the threshold value;
- The same applies for models created to assess whether an agricultural soil is eligible for sewage sludge application based on its Cu, Cd, and Zn concentrations.

## Figures and Tables

**Table 1:** The minimum, maximum, mean, median, and standard deviation (SD) of cation exchange capacity (CEC) at pH 7 (cmol_c_ kg^−1^) for base saturation (%), soil organic matter (SOM) (%), clay content (%), and pH in the topsoil samples of arable land in Sweden used in the analyses (n = 1520).

	Minimum	Maximum	Mean	Median	SD
CEC	3	70	17	15	8
Base saturation	8	100	69	72	21
SOM	0.8	16.6	4.5	4.2	1.8
Clay content	2	80	23	19	15
pH	4.5	8.4	6.2	6.2	0.6

**Table 2:** Descriptive statistics of the elements used for modelling after removal of samples with “not a number” (NaN) classification in any of the elements included (n = 1520). Minimum, maximum, mean, median, and standard deviation (SD) are presented as mg kg^−1^, where values < 1000 were rounded to the closest integer and values > 1000 to three significant digits. Rec = mean recovery rates from four measurements based on reference standard 2709a from the National Institute of Standards and Technology (NIST) (%); Rec-SD = standard deviation of the four recovery rates (%).

Element	Minimum	Maximum	Mean	Median	SD	Rec	Rec-SD
Pb	8	146	19	18	7	63	10.8
Cs	10	56	33	34	9	970	33.1
Zn	16	518	72	67	32	92	2.1
V	33	411	93	90	30	123	18.6
Rb	32	181	104	100	26	83	0.8
Sr	71	378	142	132	49	92	0.8
Zr	71	955	251	240	77	65	0.9
Ba	197	1140	491	487	98	87	2.4
Mn	124	6000	542	481	345	97	2.7
Ti	1630	6890	3860	3880	765	114	1.8
Ca	2980	196,000	11,100	9710	9390	105	1.5
Fe	4370	93,000	21,500	19,300	9760	84	0.6
K	11,400	36,200	24,100	24,300	4180	96	1.3

**Table 3:** Descriptive statistics of lab-analyzed copper (Cu), zinc (Zn), and cadmium (Cd) for the calibration data (national dataset, n = 1520) and validation data (farm dataset, n = 179). Minimum, maximum, mean, median, and standard deviation (SD) are presented as mg kg^−1^ rounded to the closest integer, apart from those for Cd.

Lab-Analyzed Element	Minimum	Maximum	Mean	Median	SD
**National dataset**					
Cu	2	130	14	11	10
Zn	6	557	61	56	33
Cd	0.04	4.07	0.20	0.17	0.17
**Farm dataset**					
Cu	3	77	22	17	19
Zn	22	135	72	67	30
Cd	0.06	1.60	0.37	0.21	0.38

**Table 4:** Validation statistics from the cross-validation of the multiple linear regression (MLR), random forest regression (RF), and multivariate adaptive regression spline (MARS) models for copper (Cu), zinc (Zn), and cadmium (Cd). R^2^ = coefficient of determination; MAE = mean absolute error (mg kg^−1^); ROI = range of interest (0–20 mg kg^−1^ for Cu and 0–0.5 mg kg^−1^ for Cd).

Model	R^2^	MAE	R^2^-ROI	MAE-ROI
Cu-MLR	0.58	3.87	0.06	3.00
Cu-RF	0.63	3.48	0.20	2.69
Cu-MARS	0.59	3.72	0.04	2.94
Zn-MLR	0.92	5.60	-	-
Zn-RF	0.86	5.93	-	-
Zn-MARS	0.92	5.63	-	-
Cd-MLR	0.49	0.065	−0.17	0.057
Cd-RF	0.48	0.053	0.40	0.043
Cd-MARS	0.70	0.054	0.20	0.047

**Table 5:** Validation statistics from the farm dataset of the multiple linear regression (MLR), random forest regression (RF), and multivariate adaptive regression spline (MARS) models for copper (Cu), zinc (Zn), and cadmium (Cd). R^2^ = coefficient of determination; MAE = mean absolute error (mg kg^−1^); ROI = range of interest (0–20 mg kg^−1^ for Cu and 0–0.5 mg kg^−1^ for Cd).

Model	R^2^	MAE	R^2^-ROI	MAE-ROI
Cu-MLR	0.90	4.40	0.12	3.56
Cu-RF	0.84	4.51	0.54	2.43
Cu-MARS	0.94	3.21	0.47	2.72
Zn-MLR	0.96	4.40	-	-
Zn-RF	0.94	5.40	-	-
Zn-MARS	0.97	4.00	-	-
Cd-MLR	0.74	0.121	0.34	0.052
Cd-RF	0.74	0.109	0.44	0.050
Cd-MARS	0.80	0.087	0.50	0.043

**Table 6:** Confusion matrices for classifications above and below thresholds for copper (Cu) fertilization and sewage sludge application for Cu, zinc (Zn), and cadmium (Cd) using the best models for each element in the cross-validation. Swedish recommendations suggest that there is risk of Cu deficiency if the Cu concentration in the soil is below 8 mg kg^−1^, while sewage sludge application is prohibited if the concentrations of Cu, Zn, and Cd exceed 40, 100, and 0.4 mg kg^−1^, respectively.

Cu Fertilization		Lab-Analyzed		Total
		Below Threshold	Above Threshold	
**Predicted**	**Below Threshold**	224	70	294
	**Above Threshold**	200	1026	1226
**Total**		424	1096	
**Cu Sewage Sludge**		**Lab-Analyzed**		**Total**
		**Below Threshold**	**Above Threshold**	
**Predicted**	**Below Threshold**	1490	27	1517
	**Above Threshold**	2	1	3
**Total**		1492	28	
**Zn Sewage Sludge**		**Lab-Analyzed**		**Total**
		**Below threshold**	**Above Threshold**	
**Predicted**	**Below Threshold**	1337	21	1358
	**Above Threshold**	44	118	162
**Total**		1381	139	
**Cd Sewage Sludge**		**Lab-Analyzed**		**Total**
		**Below Threshold**	**Above Threshold**	
**Predicted**	**Below Threshold**	1437	49	1486
	**Above Threshold**	18	16	34
**Total**		1455	65	

## Supplementary Materials

The following are available online at https://www.mdpi.com/1424-8220/20/2/474/s1: data from PXRF measurements on reference standards with the PXRF device used in this study and equations for the MLR models.
